# Individuals under voluntary treatment with sexual interest in minors: what risk do they pose?

**DOI:** 10.3389/fpsyt.2023.1277225

**Published:** 2023-11-23

**Authors:** Fritjof von Franqué, Ralf Bergner-Koether, Stefanie Schmidt, Jan S. Pellowski, Jan H. Peters, Göran Hajak, Peer Briken

**Affiliations:** ^1^Institute for Sex Research, Sexual Medicine and Forensic Psychiatry, University Medical Center Hamburg-Eppendorf, Hamburg, Germany; ^2^Department for Sexual Medicine, Sozialstiftung Bamberg, Bamberg, Germany; ^3^Department of Clinical Psychology and Psychotherapy, University of Bamberg, Bamberg, Germany; ^4^Department of Educational Psychology, University of Bamberg, Bamberg, Germany

**Keywords:** child sexual abuse, child pornography, Dunkelfeld, *do not offend*, not become an offender, kein täter werden, CPORT

## Abstract

Child Sexual Abuse (CSA) and the production, use, and distribution of Child Sexual Abuse Material (CSAM) are key threats to children’s mental health. From the perspective of indicated prevention, it can be assumed that some persons with a sexual interest in children commit such unreported crimes. Accordingly, the German Network *kein Täter werden* (meaning *do not offend*) has implemented a confidential treatment service for persons with a sexual interest in minors who voluntarily seek therapy, might or might not have offended but have not yet been detected or have fulfilled all legal requirements (here referred to as non-forensic individuals). However, this offer has been questioned for investing resources in a group which critics consider as low risk. The following study addresses the question of recidivism risks for CSA or viewing CSAM among non-forensic individuals. We found significantly higher rates of CSA/CSAM in our participants’ history compared to a German study on a representative sample of males. Regarding CSAM, the recidivism rate of 39% was found to be 11 times higher than the expected recidivism rate based on previous publications. Regarding CSA, the recidivism rate of 14% was not significantly different from the expected rate reported for subjects with a conviction for a sexual contact offense. Among various risk instruments, only the CPORT with CASIC rating was able to predict CSA (AUC = 0.69, 95% CI = 0.55, 0.82) and CSAM (AUC = 0.63, 95% CI = 0.53, 0.73) among individuals with a history of CSAM, but with poor discrimination. We conclude that a large proportion of our sample poses a substantial risk and therefore treatment resources are well invested. However, further studies are needed to improve risk assessment among non-forensic clients.

## 1 Introduction

Sexual violence, especially sexual violence against children, is a significant worldwide problem. Two meta-analyzes involving over 9.9 million participants in six continents found lifetime prevalences for child sexual abuse (CSA) of 18–20% for girls and 8% for boys (1, 2). Lifetime prevalence estimates, using representative sampling, show that in a sample of 8,718 German men, 1.7% reported having seen child sexual abuse material (CSAM) before, 0.8% said they had abused at least one child and 0.7% said they had done both (3). In addition, the official police crime statistics indicate a rise in the detected cases of sexual violence against children: for example, in 2021, the numbers for CSA in Germany increased by 6.3% to more than 15,500 cases, while the use of CSAM increased by 108.8% to more than 39,000 cases. Similar results can be found for other countries. Although the increase might partly be due to changes in the legal system, it can also be assumed that more and more offenses are being reported. However, the overall number of unreported crimes is still many times higher (4): one of the above-mentioned meta-analyzes (2) showed that the prevalence rate of CSA in self-report studies was 12.7%, while a CSA prevalence rate of only 0.4% resulted in so-called informant studies (i.e., “reports of professionals, dossier or chart reviews, and informant observations of children such as teachers observing their students in primary schools,” p. 80).

From the perspective of indicated prevention, this raises the question of which individuals commit unreported offenses and whether they can be reached therapeutically. Looking at the meta-analysis by Hanson and Morton-Bourgon (5), it can be assumed that a substantial number of persons with a history of CSA showed a sexual interest in minors, as sexual deviance was shown to be a strong predictor of reoffending. Accordingly, Beier et al. (6) concluded that treatment services should be provided especially for those individuals with a pedo-hebephilic sexual interest in children. As a consequence, the network *kein Täter werden* (which means *do not offend*) implemented a confidentiality-protected treatment offer since the year 2005. Treatment is offered to individuals who (a) are concerned about committing CSA or using CSAM, (b) have a history of committing CSA or using CSAM, but wish to discontinue this behavior, and are not known to authorities, and (c) have been convicted of CSA or CSAM, but have completed all legal matters, and continue to need further treatment. We refer to these individuals as non-forensic group. The network includes 13 different locations spread across the Federal Republic of Germany. First evaluation results regarding the treatment were published by Beier et al. (6), Kuhle et al. (7), and Franqué and Briken (8). The different authors concluded that dynamic risk factors could be reduced by the treatment and thus the network could make a significant contribution to the prevention of child sexual abuse.

**Figure 1:**
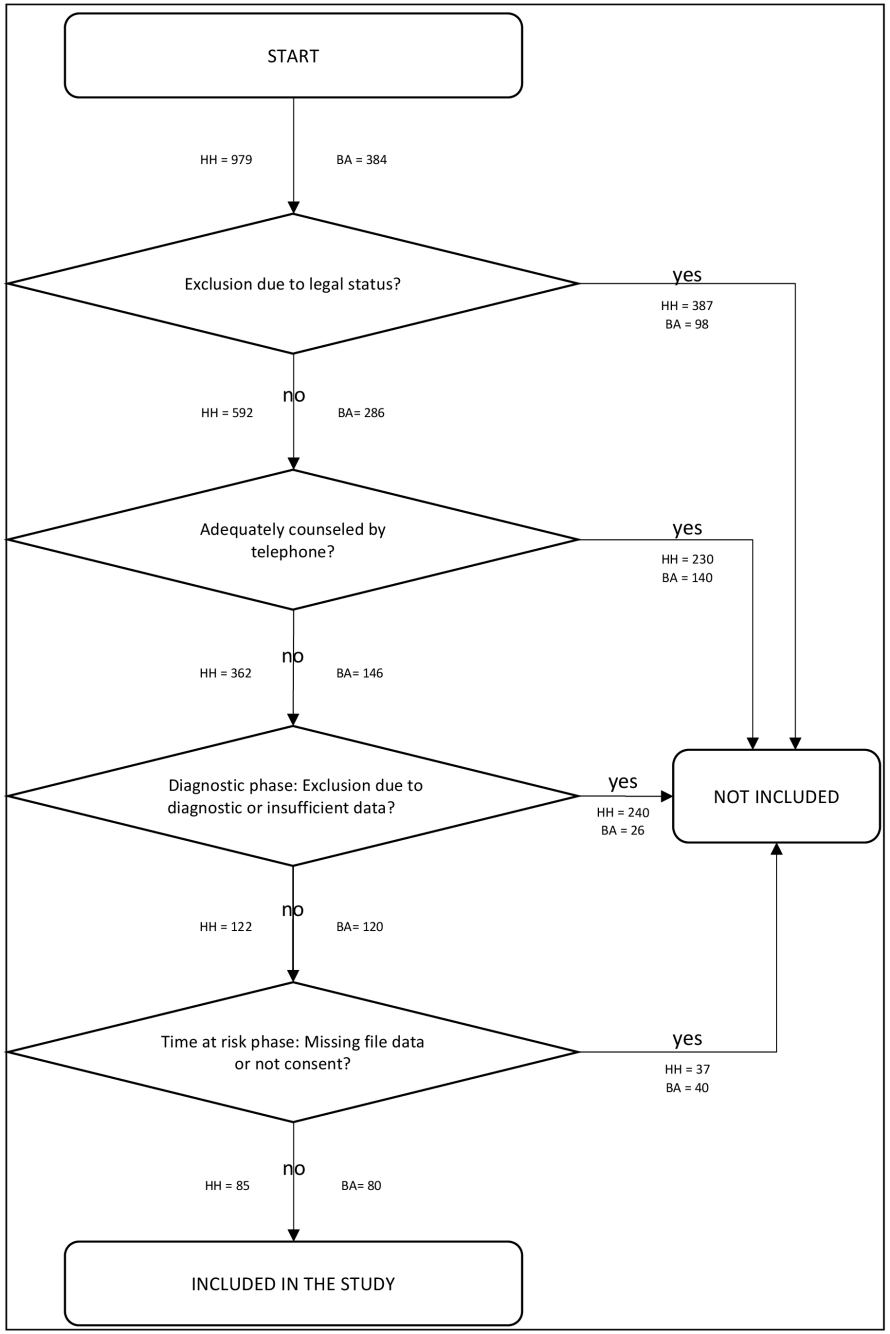
Inclusion process of the study; HH, HAMBURG/Germany; BA, BAMBERG/Germany.

However, these assumptions and conclusions have also been criticized: for example, the evaluation study by Beier et al. (6) was criticized for including only an insufficient number of dynamic risk factors, for having an inadequate methodological approach, and for not being able to demonstrate treatment effects with adequate statistical procedures (9–11). In addition, König (12, p.119, translated from the original German work) argued: ‘Instead of expanding primary preventive services, the effectiveness of which has yet to be proven in terms of child and youth protection, it would make sense from a forensic perspective to expand the outpatient psychotherapeutic care situation for men who have been convicted of sexual offenses against minors and have received court orders for therapy, for example. This is a group of offenders who have already proven their dangerousness and, in accordance with the RNR-principle, have a particular need for help. In particular, it was argued that a risk for reoffending exists for individuals who had been sanctioned, rather than for undetected participants or individuals without problematic behavior at all (H. -L. Kröber, personal communication, January 9th, 2015). According to this critique, the difference between reported and self-reported offenses is more likely to be viewed through detection evasion skills (13) of those individuals who sooner or later come in conflict with the law. According to this argument, treatment resources should rather be allocated to individuals who are at a higher risk, which is in line with the risk-need-responsivity model of rehabilitation (14). Hart et al. (15) define the term risk as “a hazard that is incompletely understood and whose occurrence therefore can be forecast only with uncertainty.” From our perspective, risk is mostly operationalized using recidivism rates of individuals who have already committed (sexual) crimes: Helmus et al. (16) summarized the empirical situation regarding sexual contact offending in a short review with 73 studies and 35,522 included subjects: From their perspective, most studies conclude recidivism rates between 10% and 15%. A mean follow-up period is not given, but according to Figure 1 in the mentioned publication, the time at risk period for the corresponding studies seems to vary between 1 year and 27 years. For CSAM recidivism, Seto et al. (17) report a rate of 3.4% during a 1.5- to 6-year follow-up period. Another way to determine the risk of individuals is to use validated risk assessment instruments. These include, for example, the STATIC-99R (18) or The Child Pornography Offender Risk Tool [CPORT; (19)]. One problem with both operationalizations is that they are oriented toward recorded offenses and take less into account the occurrence of sexual violence *per se*, even when it has not been officially charged. In this respect, there has been a lack of validation studies for risk assessment instruments based on incidents of sexual violence, independent from legal detection.

In line with these preceding considerations, our paper addresses the following question: What is the risk for CSA and CSAM posed by individuals who presented themselves voluntarily and without a treatment requirement in the context of the network *do not offend*? How can we assess risk in this context? Based on the information provided by the study participants, we first compared the rates of CSA and CSAM in the history of the study participants with the prevalence rates in a representative German sample (3). Second, we contrasted the recidivism rates of CSA and CSAM of our sample with recidivism rates as reported by Helmus et al. (16) for CSA and by Seto et al. (17) for CSAM. Third, we investigated the results of the risk assessment instruments STATIC-99, STATIC-C, and the CPORT and tested their predictive validity.

## 2 Method

### 2.1 Background

The network *kein Täter werden* (which means *do not offend*) includes 13 different locations spread across the Federal Republic of Germany. The different institutions are not united by a uniform therapeutic approach [*cf.* (20, 21)], but by certain quality standards, such as the fact that therapists have started or completed training in psychotherapy and sex therapy. In addition, all sites base their treatment on the known dynamic risk factors for sexual recidivism with a special focus on pedophilic disorder (22), meaning sexual impulses and fantasies with children in the prepubescent (pedophilia) or early stages of puberty (hebephilia) persisting for at least 6 months in combination with clinically significant distress or sexual problematic behavior based on these fantasies or impulses (23). We refer to this diagnosis as pedo-hebephilic disorder in the following. The work of the network is aligned with the special conditions of German jurisdiction: Accordingly, therapists must adhere to medical confidentiality and not disclose information about a perpetrated CSA or possession of CSAM in their clients’ past unless there is an acute risk for sexual violence. More information about the network can be found at www.kein-taeter-werden.de or www.troubled-desire.com/en/.

### 2.2 Measures

#### 2.2.1 STATIC-C

The STATIC-C is a custom-made measure for assessing the static risk in individuals with a history of CSA or CSAM or who are at risk for these behaviors. The STATIC-C is used to allocate therapeutic resources according to the risk-principle of the risk-need-responsivity model [RNR; (14)]. The instrument may be used if only self-reported information to be assessed is available and further information (e.g., a criminal register) is missing. The STATIC-C was designed on the basis of the STATIC-99 (24). The measure includes 12 items, 9 of which are answered on a scale of 0 to 1. Two additional items are answered on a scale between 0 and 2, and one item is coded between 0 and 3. The items contain the client’s age (1 = older than 25 years), relationship history (1 = never in a relationship of 2 years), nonsexual violence (1 = at least one reported incident of actual, attempted, or threatened harm to another person), reported prior convictions, ICD-10 diagnosis of pedo-hebephilic disorder (1 = pedo-hebephilic disorder, non-exclusive type, 2 = pedo-hebephilic disorder, exclusive type), other paraphilic disorders (1 = paraphilic disorder except sadistic disorder, 2 = sadistic disorder) and personality disorders (1 = any personality disorder), prior use of CSAM (1 = prior use of CSAM), as well as the number (1 = two different persons, 2 = three different persons, 3 = four or more different persons) and sex (1 = male) of individuals harmed plus their relationship (1 = strangers to each other; 1 = unrelated) with the person being assessed. Accordingly, a total sum score between 0 and 16 points is obtained. Values between 0 and 2 are deemed to indicate a low risk of relapse. Scores between 3 and 4 indicate a low to average risk of relapse, between 5 and 6 an average to high risk, and those of 7 and more finally a high risk.

The validity and reliability of the STATIC-C was examined in a study by Kalt (25): reliability coefficients between 0.56 and 1.0 (Cohen’s Kappa) resulted for the 12 items, while a coefficient of 0.96 (ICC) resulted for the total sum score. Concerning the predictive validity, an AUC value of 0.74 was obtained at a recidivism rate of 3.8%, which, however, did not become statistically significant. The STATIC-C was also used in a study by von Franqué and Briken (8) to compare the static risk of forensic and non-forensic clients.

#### 2.2.2 STATIC-99

The STATIC-99 is designed to measure the static risk for sexually motivated reoffending among adult males who have previously been charged with or convicted of a contact sex offense (24). The instrument was completely revised in 2003 (26) and translated into various languages [e.g. German Version: (27)]. The revised version is used in the present study because, according to a paper by Eher et al. (28), the measures used were found to be more valid for samples from German-speaking countries compared to the even more recent form, the STATIC-99R (18).

The instrument includes 10 exclusively static risk factors, of which 9 items can be scored between 0 or 1, and one item is coded with a score between 0 and 3. Accordingly, the total sum score of the measure varies between 0 and 12 points. The items determine whether the person being assessed is younger than 25 years of age, never had a relationship of more than 2 years, has a criminal and especially sexual offense history, and finally has harmed an unknown, male, and unrelated person through sexual violence. Here, a value of 0 indicates a low-risk level. After that, the other categories break down into below-average (1 and 2), average (3 and 4), above-average (5 and 6), and finally significantly above-average (7 and more) risk of relapse (26).

The STATIC-99 is one of the best-studied risk-assessment instruments. According to various reviews of its psychometric properties, interrater reliability was found to be acceptable in most studies (> 0.75 in almost all studies), while predictive validity was found to be moderate to high [e.g., fixed-effect AUC = 0.68, 95% CI = 0.67, 0.69; random-effects AUC = 0.69, 95% CI =0.67, 0.71, *k* = 56, *n* = 71,515; (29)].

#### 2.2.3 CPORT

The Child Pornography Offender Risk Tool [CPORT; (19)] is an instrument for assessing the static risk for any sexual recidivism (meaning contact sexual offenses and non-contact sexual offenses; non-contact sexual offenses include child pornography delinquency as well as offenses such as indecent exposure) among adult males with a conviction for child pornography offenses (19). A German translation is available (30). The CPORT consists of 7 items. All items are scored on a scale between 0 (= absent) and 1 (= present). The items contain (1) offender aged 35 or younger at time of index investigation; (2) any prior criminal history; (3) any failure on conditional release, whether bail, probation or parole; (4) any contact sexual offending; (5) indication of pedophilic or hebephilic sexual interests, that is, pertaining to prepubescent or pubescent children; (6) more boy than girl content in child pornography seized by police; and, (7) more boy than girl content in other child-related content. Accordingly, the total value of the measure can vary between 0 and 7 points. Scores of 0 and 1 correspond to low, 2 and 3 to low to moderate, 4 to moderate to high, and 5 and above to high risk of relapse (31).

According to a study by Seto and Eke (19), CPORT score was a moderately strong predictor of any sexual recidivism (AUC = 0.74, 95% CI =0.63, 0.84).

##### 2.2.3.1 Casic

In the absence of a pedo-hebephilic diagnosis, the CPORT Item 5, indication of pedophilic or hebephilic sexual interests, can be assessed using the Correlates of Admission of Sexual Interest in Children (CASIC) scale. This scale consists of 6 items, which are coded with 0 (= no) or 1 (=yes). The content asks whether (1) someone has never been married, (2) has possessed child pornography material, (3) has possessed child pornography writings, (4) has had an interest in child pornography for at least 2 years, (5) holds a volunteer position with close contact with children, and (6) has had online communication of a sexual nature with a minor. Accordingly, a total score between 0 and 6 points may result, with a score of ≥3 considered to indicate the presence of pedo-hebephilic interests.

In the development sample, the CASIC total score with an AUC-value of 0.71 was significantly associated with admission of sexual interest in children. In a cross-validation sample an AUC value of 0.81 resulted between the CASIC total score and the admission of sexual interest in children (32).

### 2.3 Participants

The present study consists of a total sample of 165 persons, 80 from Bamberg and 85 from Hamburg, who were selected from two locations from the network *do not offend*. In the following, the selection process is described in more detail for the respective locations. A summary of the selection process is shown in Figure 1.

#### 2.3.1 Hamburg

Between April, the 1st of 2012, and November, the 9th of 2021, 979 interested persons sought contact with the project’s office by telephone or e-mail. In 223 cases, CSA or viewing CSAM was already known to the authorities before contact was made, thus resulting in exclusion from the prevention project. In addition, the legal status of 164 cases remained unclear because these individuals used an e-mail for initial contact but did not comply with our written request for a follow-up phone call. Of the remaining 592 persons, 230 could adequately be counseled by telephone. For example, we referred persons to more accessible or appropriate treatment services (e.g., for problems with adolescents or stalking). In 53 cases, the individuals could not be reached at all for appointments based on their information. The remaining 309 individuals were invited for an initial interview. Of these, 33 individuals dropped out during the diagnostic process. 88 persons went through a short diagnostic process and were excluded afterward. Reasons were a young age (< 23 years due to an alternative offer for adolescents and young adults with a pedo-hebephilic interest in Hamburg), acute substance problems, psychotic or obsessive-compulsive symptoms (related to pedo-hebephilic impulses), acute suicidal thoughts and impulses. Predominantly, individuals with substance use problems or with obsessive-compulsive disorder, who thought they had pedo-hebephilic interest, were excluded. 57 individuals completed the project’s comprehensive diagnostic process but were not included because these participants only wanted a thorough assessment of their sexual interests. In the case of 9 persons, the diagnostic process had not yet been completed. Thus, 122 individuals were included. Of these participants, only 85 individuals gave informed consent for a more detailed analysis of their data. At the time of the study, 29 persons were still in therapy, while one participant had completed the diagnostic phase and a follow-up interview only. 51 individuals had already completed the treatment program. 4 subjects dropped out during treatment. 15 participants had a follow-up interview, with the follow-up period ranging from 9 to 68 months.

#### 2.3.2 Bamberg

The site is well connected and very closely located to the psychiatric clinic so that individuals suffering from psychiatric symptoms (psychotic disorder, acute substance problems, or suicidal impulses) were not immediately excluded. They were brought to the attention of the psychiatric clinic, and returned to the program after hospital discharge if they still fulfilled the program’s criteria. Individuals with obsessive-compulsive disorder were included if they reported seeing CSAM in their past to “test” a sexual interest in children. If they did not meet the criteria for pedophilic disorder or did not pose a risk for CSAM, they were excluded from the program and informed about other treatment options. Thus, the ratio of individuals being excluded is less than in Hamburg. From December 2015 to November 2021, 384 persons contacted the site in Bamberg, Germany. Apart from the already mentioned differences, the selection process was similar to the one in Hamburg. 98 persons were excluded due to their legal status, while another 140 felt already adequately counseled by telephone. After the initial diagnostic phase another 26 people could not be included, so that only 120 persons were offered treatment. Four of those were excluded due to too much missing data or they did not give informed consent. 36 participants did not show up for a second appointment and were thus excluded. Three subjects were in an extended diagnostic phase with some elements of psychoeducation but had not yet started actual therapy. During the treatment process, 35 individuals dropped out of the program before officially finishing the therapy due to various reasons (moving, not showing up anymore, getting a new job and not being able to make time anymore). Some of these individuals had already attended therapy for a longer period and therapy was about to end. At the time of the study, 25 persons participated in the treatment program. 16 individuals had already completed the program. Nine individuals participated in a follow-up interview, with a follow-up period ranging from two to approximately 36 months.

### 2.4 Procedure

#### 2.4.1 Diagnostic phase

All subjects included in the present study underwent a semi-structured interview lasting several hours and completed a series of questionnaires. External sources such as criminal records or hospital reports were usually not available and could not be taken into consideration. Most participants did not agree to request such documents because they were concerned about their privacy. The case information included socio-demographic and biographic data, sexual history, ICD-10 diagnoses, information about committing CSA and possessing CSAM, charges or convictions of CSA or CSAM (hereinafter referred to as “detected”), and risk-assessment data from the STATIC-C and other instruments not used in the given study. The case information existing at the time of admission formed the basis for the assessment of the risk assessment instruments STATIC-99 and CPORT, which were rated retrospectively. The files of the participants were evaluated by five raters from two different sites.

#### 2.4.2 Time at risk phase

After the end of the diagnostic phase, the participants entered the evaluation phase, which is referred to as time at risk. This includes the therapeutic course and follow-up interviews after the end of therapy. The files of the participants were evaluated by 5 raters regarding the occurrence of CSA and CSAM, based on session protocols, session questionnaires, and annual risk assessments.

For the present study, we defined CSA following the definition of Hart et al. (15), as sexually motivated contact with at least one child (younger than 14 years) who cannot consent to the contact. This definition also includes acts without direct touching, such as masturbation in front of a child or instructing a child to perform sexual acts on himself or others. We examined follow-up data according to whether CSA was reported during the course. In Hamburg, the interrater reliability could not be calculated, as none of the subjects in the random sample of 12 was found to have had an incident of CSA (all three raters had concordantly assessed no incidents of CSA). For Bamberg, in a random sample of 10 individuals, the interrater reliability of two raters was 1.00 (Cohen’s Kappa), implying an almost perfect agreement according to Landis and Koch (33).

CSAM was defined as photorealistic material in which at least one child (1) is depicted in an unnatural, sexualized manner, (2) performs acts on himself or herself that are understood to be sexual, or (3) performs or is induced to perform acts with at least one other person that are understood to be sexual. Time at risk data were coded according to whether CSAM was used. For Hamburg, in a random sample of 12 subjects, the interrater reliability of three raters was 0.86 (Fleiss Kappa) for CSAM, implying an almost perfect agreement according to Landis and Koch (33). For Bamberg, in a random sample of 10 individuals, the interrater reliability of two raters was 0.73 (Cohen’s Cappa), implying a substantial agreement according to Landis and Koch (33).

### 2.5 Design

The present study used a static group design (34) by comparing the relative frequencies of CSAM and CSA in the present sample with (1) the relative frequencies of CSAM and CSA in a representative German study (3) and (2) the recidivism rates of CSA (16) and CSAM (17) of previously sentenced individuals.

In terms of predictive validity, our study used a retrospective cohort design. The Static-99 and CPORT were rated using case information available at the end of the diagnostic phase. In contrast, the data on STATIC-C were already part of the diagnostic phase. The occurrence of CSAM and CSA was rated using all available information during the time-at-risk phase.

### 2.6 Statistics

For comparisons of relative frequencies, we used the exact *Binomial test*. As effect sizes, we also calculated relative risk, defined as the observable probability divided by the expected probability. For the calculation of relative risks, we used the unrounded probabilities from our sample and from the corresponding publications by Dombert et al. (3), Helmus et al. (16) or Seto et al. (17), whereby we also calculated confidence intervals in the case of available absolute values.

For the association between the occurrence of CSA/CSAM and the sum scores of the risk assessment instruments Static-99, STATIC-C, and CPORT, we calculated point-biserial correlations. *Receiver operating characteristic* (ROC) curves were determined in addition and the *area under the curve* (AUC) was calculated to assess how well the different risk assessment measures predict CSA/CSAM during time at risk. As predictors, we used the total scores of the risk assessment instruments STATIC-99, STATIC-C, CPORT with clinical diagnosis by expert raters for CPORT item 5 (indication of pedophilic or hebephilic interests) and CPORT with CASIC rating for CPORT item 5. As dependent variables, we selected (a) self-reported viewing of CSAM and (b) self-reported committing CSA. Moreover, we analyzed data including all participants of our study and data including only participants that had a history with the respective sexual problematic behavior (CSA or CSAM, respectively). For the analysis of the subsamples, we restricted the computations to the measures that claimed validity for the respective subsample (CPORT for individuals with a history of CSAM, STATIC-99 for participants with a history of CSA, and STATIC-C for persons with a history of CSA/CSAM).

## 3 Results

Table 1 gives an overview of the descriptive data in the sample.

**Table 1:** Descriptive data from 165 non-forensic clients with sexual interest in children, distinguished by sexual problem behaviors in their history.

Mean (SD)/Number(Percent)/Median (Range)	Group				
	No offense (*n* = 17)	CSAM^a^ only (*n* = 90)	CSA^b^ only (*n* = 16)	Mixed^c^ (*n* = 42)	Total (*n* = 165)
*Sociodemographic data*					
Age in years (SD)	31.06 (7.90)	34.21 (11.56)	44.44 (16.05)	39.21 (10.76)	36.15 (12.04)
Male subjects	15 (88%)	89 (99%)	16 (100%)	42 (100%)	162 (98%)
More than 10 years in school	10 (59%)	67 (74%)	8 (50%)	26 (62%)	111 (67%)
With a job	11 (65%)	65 (72%)	11 (69%)	31 (74%)	118 (72%)
With an intimate relationship	4 (24%)	39 (43%)	9 (56%)	26 (62%)	78 (47%)
With children	2 (12%)	9 (56%)	8 (50%)	20 (48%)	49 (30%)
Living alone	7 (41%)	44 (49%)	8 (50%)	14 (33%)	73 (44%)
*Diagnostic data*					
With ICD-10 pedophilic disorder	10 (59%)	84 (93%)	9 (56%)	42 (100%)	145 (88%)
With CASIC-Score > 2	2 (12%)	48 (53%)	3 (19%)	21 (50%)	74 (45%)
With ICD-10 pedophilic disorder and CASIC-Score > 2	2 (12%)	45 (50%)	2 (13%)	21 (50%)	70 (42%)
With any other ICD-10 paraphilia	4 (24%)	16 (18%)	5 (31%)	11 (26%)	36 (22%)
With hypersexual disorder	1 (6%)	10 (11%)	0 (0%)	2 (5%)	13 (8%)
With any personality disorder	4 (24%)	12 (13%)	3 (19%)	9 (21%)	28 (17%)
With any affective disorder	2 (12%)	30 (33%)	1 (6%)	10 (24%)	43 (26%)
*Forensic data*					
Previous conviction for CSAM	0 (0%)	14 (16%)	0 (0%)	7 (17%)	21 (13%)
Previous conviction for CSA	0 (0%)	0 (0%)	4 (25%)	8 (19%)	12 (7%)
*Risk-assessment data*					
Months at risk (SD)	22.70 (23.79)	28.47 (24.30)	23.90 (20.21)	28.32 (22.21)	27.40 (23.25)
STATIC-99 score (SD)	1.18 (0.64)	1.21 (1.11)	1.69 (1.96)	2.10 (1.43)	1.48 (1.31)
STATIC-99 median (range)	1.00 (2.00)	1.00 (4.00)	1.00 (5.00)	2.00 (7.00)	1.00 (7.00)
STATIC-C score (SD)	2.65 (1.84)	3.48 (1.66)	3.94 (2.86)	6.02 (2.07)	4.08 (2.24)
STATIC-C median (range)	3.00 (8.00)	3.00 (10.00)	3.00 (9.00)	6.00 (9.00)	4.00 (11.00)
CPORT score (SD)	1.65 (1.22)	2.19 (1.04)	1.81 (1.38)	2.45 (1.15)	2.16 (1.14)
CPORT median (range)	1.00 (4.00)	2.00 (5.00)	1.50 (5.00)	2.00 (4.00)	2.00 (5.00)
*Sexual problematic behaviors during time at risk*					
Child sexual abuse material	0 (0%)	34 (38%)	1 (6%)	18 (43%)	53 (32%)
Child sexual abuse	1 (6%)	0 (0%)	3 (19%)	5 (12%)	9 (5%)

^a^Participants with Child Sexual Abuse Material in their past, ^b^Participants with Child Sexual Abuse in their past, ^c^Participants with Child Sexual Abuse and Child Sexual abuse Material in their past.

### 3.1 History of CSA and CSAM

According to Table 1, of all the people participating in the diagnostic procedure the observed frequency of lifetime CSA was 35% (see Table 1: CSA only + Mixed), whereas the expected lifetime prevalence according to the representative study by Dombert et al. (3) would be 1.5%. In the exact binomial test, there was a significant difference between the expected frequency of 0.015 and the observed frequency of 0.35 (*p* < 0.001, 1-sided) with a relative risk of 23.2 (95% CI = 17.77; 30.34).

The observed frequency of CSAM (see Table 1: CSAM only + Mixed) in the participants’ history was 80%, whereas the expected lifetime prevalence in the representative sample of Dombert et al. (3) would be 2.4%. In the exact binomial test, a significant difference between the expected frequency of 0.024 and the observed frequency of 0.80 resulted (*p* < 0.001, 1-sided) with a relative risk of 33.4 (95% CI =28.6; 38.9).

### 3.2 CSA and CSAM during time at risk

Table 2 lists the rates of CSA reported by subjects during the time at risk phase.

**Table 2:** Frequencies (percent within group) of child sexual abuse (CSA) during time at risk, differentiated by detection of CSA offending in history.

CSA in history	No offense (*n* = 107)	1 (1%)
	Undetected CSA (*n* = 46)	8 (17%)
	Detected CSA (*n* = 12)	0 (0%)
Total (*n* = 165)		9 (5%)

For the statistical comparison of observed and expected frequencies of CSA recidivism, only the 58 individuals, who had committed CSA in the past (see Table 1: CSA only + Mixed; see Table 2: undetected CSA + detected CSA), were included. The observed frequency of CSA recidivism during time at risk was 14%, whereas the expected frequency according to the review of Helmus et al. (16) should be 15% in individuals with a detected sexual offense. According to the exact binomial test, there was no statistical difference between the observed frequency of CSA of 0.14 and the expected frequency of 0.15, *p* = 0.49 (1-sided). A relative risk of 0.92 (95% CI not available) resulted.

Table 3 shows the frequencies of CSAM reported by subjects during the time at risk phase.

**Table 3:** Frequencies (percent within group) of persons with child sexual abuse material (CSAM) during time at risk, differentiated by detection of CSAM use in history.

CSAM in history	No CSAM (*n* = 33)	1 (3%)
	Undetected CSAM (*n* = 111)	49 (44%)
	Detected CSAM (n = 21)	3 (14%)
Total (*n* = 165)		53 (32%)

For the statistical comparison of observed and expected frequencies of CSAM recidivism, only the 132 participants who had used CSAM in their history (see Table 1: CSAM only + Mixed; see Table 3: undetected CSAM + detected CSAM) were selected. The observed frequency of CSAM recidivism during time at risk was 39%, whereas the expected frequency of detected CSAM users according to the review by Seto et al. (17) would be 3.4%. In the exact binomial test, a significant difference between the expected frequency of 0.034 and the observed frequency of 0.39 (*p* < 0.001, 1-sided) with a relative risk of 11.42 (95% CI =7.95, 16.41) resulted.

### 3.3 Risk assessment

#### 3.3.1 Risk for CSA

Analyzes including all participants revealed no significant association between CSA during time at risk with the total sum scores of the STATIC-99 (r = 0.06, *p* = 0.48), the STATIC-C (r = 0.07, *p* = 0.34), the CPORT with clinical diagnosis (r = 0.09, *p* = 0.27) and the CPORT with CASIC rating (r = 0.08, *p* = 0.32). Accordingly, no significant AUC resulted with the STATIC-C (AUC = 0.61, 95% CI = 0.40, 0.83, *p* = 0.31), the STATIC-99 (AUC = 0.58, 95% CI = 0.39, 0.76, *p* = 0.42), the CPORT with clinical diagnosis (AUC = 0.59, 95% CI = 0.40, 0.79, *p* = 0.34) and the CPORT with CASIC rating (AUC = 0.59, 95% CI = 0.40, 0.78, *p* = 0.35).

When restricting analyzes to the 58 participants with a history of CSA (see Table 1: CSA only + Mixed), no significant association resulted between CSA recidivism with the total sum scores of the STATIC-99 (r = −0.03, *p* = 0.84) or the STATIC-C (r = −0.07, *p* = 0.59). Accordingly, the AUC values from the STATIC-C (AUC = 0.46, 95% CI = 0.25, 0.68, *p* = 0.74) and the STATIC-99 (AUC = 0.49, 95% CI = 0.29, 0.69, *p* = 0.95) were not significantly different from 0.50.

When restricting analyzes to the 132 participants with a history of CSAM (see Table 1: CSAM only + Mixed), no significant correlation resulted between future CSA with the total sum scores of the CPORT with clinical diagnosis (r = 0.10, *p* = 0.26) or the CPORT with CASIC rating (r = 0.09, *p* = 0.28). However in the ROC-Analysis, there was a significant association between future CSA and CPORT with CASIC rating (AUC = 0.69, 95% CI = 0.55, 0.82, *p* = 0.006), but not with the CPORT with clinical diagnosis (AUC = 0.68, 95% CI = 0.50, 0.86, *p* = 0.05).

#### 3.3.2 Risk for CSAM

Analyzes including all participants revealed no significant associations between future CSAM during time at risk and the total sum score of the STATIC-C (r = 0.13, *p* = 0.11) or STATIC-99 (r = 0.06, *p* = 0.48), but significant associations between future CSAM and the total sum score of the CPORT with clinical diagnosis (r = 0.20, *p* = 0.01) and the CPORT with CASIC rating (r = 0.27, *p* < 0.001). According to the ROC-Analysis, the STATIC-C (AUC = 0.60, 95% CI = 0.51, 0.69, *p* = 0.04), the CPORT with clinical diagnosis (AUC = 0.61, 95% CI = 0.51, 0.70, *p* = 0.03) and the CPORT with CASIC rating (AUC = 0.66, 95% CI = 0.57, 0.75, *p* = 0.001) were able to predict future CSAM, but not the STATIC-99 (AUC = 0.56, 95% CI = 0.47, 0.65, *p* = 0.21).

When the analyzes were restricted to 132 participants with a history of CSAM (see Table 1: CSAM only + Mixed), no significant correlation resulted between future CSAM with the total sum scores of the STATIC-C (r = 0.04, *p* = 0.62) and the CPORT with clinical diagnosis (r = 0.16, *p* = 0.08), but with the CPORT with CASIC rating (r = 0.24, *p* = 0.006). In the ROC-Analysis, there was a significant association between CSAM recidivism and the CPORT with CASIC rating (AUC = 0.63, 95% CI = 0.53, 0.73, *p* = 0.009), but not with the STATIC-C (AUC = 0.55, 95% CI = 0.45, 0.65, *p* = 0.32) or the CPORT with clinical diagnosis (AUC = 0.57, 95% CI = 0.46, 0.67, *p* = 0.22).

When restricting the analysis to the 58 participants with a history of CSA (see Table 1: CSA only + Mixed), no significant association resulted between future CSAM and the total sum scores of the STATIC-99 (r = −0.02, *p* = 0.91) or the STATIC-C (r = 0.17, *p* = 0.20). According to the AUC-Values, there was no significant association between future CSAM and the STATIC-C (AUC = 0.60, 95% CI = 0.45, 0.75, *p* = 0.20) or the STATIC-99 (AUC = 0.52, 95% CI = 0.37, 0.67, *p* = 0.79).

## 4 Discussion

The aim of the study was to investigate the risk for CSA and CSAM posed by persons with a sexual interest in minors, who voluntarily contact the network *do not offend*. Looking at the results of the risk assessment instruments, the various subgroups ranged from a low to moderate risk profile on average. However, except the CPORT, all instruments were limited in terms of predictive validity. The static-99 appears to be inadequate as far as our sample is concerned, given the lack of variation (see Table 1). Interestingly, the CPORT with clinical diagnosis instead of CASIC rating could not predict recidivism behavior. Likewise, Nentzl et al. (35) study with a non-forensic sample used the practitioner’s diagnosis of a pedo-hebephilic disorder instead of CASIC rating with the result that CPORT could not predict CSAM re-use (35). According to our results, on the other hand, recidivism could be predicted by the CPORT with the CASIC rating. The question therefore arises to what extent the use of the CASIC rating could be decisive for this. The CASIC was developed to identify people with pedo-hebephilic interests when the authenticity with which answers are given can be doubted (for example in forensic contexts) and was empirically linked to pedophilic disorder (32). It relies on objective data from official records and concentrates on the content of CSAM as well as shown behavior towards children. Not being able to rely on the information given by an individual and to prevent diagnosing individuals falsely with pedo-hebephilia, the CASIC needs to select more strictly. Therefore, it is possible that individuals in our sample with a pedophilic disorder, but without a preference or with a good behavioral control would not attain the suggested cut-off score of 3 or higher. However, they still would be diagnosed with pedo-hebephilia, when reporting persisting sexual impulses and fantasies with children in the prepubescent or early stages of puberty in combination with clinically significant distress or sexual problematic behavior based on these fantasies or impulses (23). Looking at our sample, only 45% fulfilled the cut-off criteria, while 88% were given the clinical diagnosis. This suggests that the diagnosis of pedo-hebephilia, which can be connected to re-offense behavior (5), may not be a good predictor on its own (36–38). Considering that the results of Nentzl et al. (35) found no other CPORT variable to be able to predict recidivism in non-forensic clients and that our findings could only predict recidivism when the CASIC rating was used, it seems that an assessment of pedo-hebephilic behavior with a wide range of variables as suggested by the CASIC could be a key to predict re-offense behavior in non-forensic individuals. It might be that the CASIC does not really assess pedo-hebephilic interest, but instead identifies a specific subgroup of individuals with a sexual interest in minors [see also (39)], them being the ones with a potential for recidivism.

With regard to CSA and CSAM, we compared our participants with a male sample of a representative German study (3). We found significantly higher rates of CSA/CSAM in the history of our participants: Only 17 subjects (10%) had no conspicuous behavior to report at all, whereas the remaining subjects had either committed CSA, had viewed CSAM, or had even exhibited both behaviors in their past. Although this result might be expected, it underlines that most individuals in our sample request treatment after committing CSA or viewing CSAM. Therefore, the main task of the network is to prevent reoffending behavior, and less often to prevent its initial occurrence. We conclude that an important group is addressed by the network’s offering. However, critics might argue that resources for treatment should be based on the potential for re-offending and not solely on past offending behaviors, implying that many individuals in the prevention project would not offend a second time. To address this criticism, we discuss recidivism risk separately for CSA and CSAM below. However, we would argue that individuals with a high-risk profile will have had a low-risk profile at some point in their life, particularly because of the static risk factors. That these risk factors can increase over time and influence each other is currently investigated in the network-based model of reoffending [NBM-RSR; van den Berg (40)]. In this respect, an important goal of the network could be to prevent low-risk profiles from increasing. There is currently a lack of empirical criteria to distinguish individuals with an increasing profile from those with a consistently low risk, who, according to the RNR-model, either do not need treatment at all, or should receive little treatment (41). More research is needed to follow individuals with a low risk over time. However, up to this point, there is no other way in clinical practice than working with the self-selection of non-forensic clients.

Regarding future use of CSAM, the results of our study show that the recidivism risk of individuals who had viewed or distributed CSAM in the past was 11 times higher than the meta-analytically determined recidivism rate of sentenced individuals with CSAM [see (17)]. Differentiating our sample by whether CSAM was (a) not present in the history, (b) undetected, or (c) sanctioned, all three groups showed at least the frequency of occurrence (see Table 3) compared with the recidivism rate reported by Seto et al. (17). In particular, the groups undetected and detected CSAM use in history had a risk that was several times higher. Accordingly, it can be concluded that our sample is at a higher risk level, which is in accordance with other findings (6, 7, 35). This would suggest that treatment resources should be made available to individuals with a history of CSAM if policymakers wish to prevent CSAM from being viewed in the future. By allocating treatment resources to non-forensic clients, instead of waiting for CSAM users to be detected, the total number of re-offense behavior might be reduced. In our sample, this concerns 80% of the participants (Table 1: CSAM only + Mixed). However, from a risk perspective and regarding the moderate predictive validity of the CPORT with CASIC rating, one would additionally wish for a measure with stronger associations with the CSAM recidivism rate. This ties back to the question of which variables in addition to the CPORT might increase predictive validity. According to Tables 2, 3, a promising predictor for future research could be the undetected occurrence of CSAM in comparison to detected CSAM. It might be that a person not having been detected, does not feel the need to change their behavior as much as an already sanctioned individual.

With regard to future occurrence of CSA, our results indicate that the recidivism rate of individuals who had a history of CSA is not significantly different from the meta-analytically determined rate of 15% for individuals with a conviction for a sexual offense (16). This implies that at least this subgroup of our study (Table 1: CSA only + Mixed) should receive similar resources as individuals with a conviction of CSA in their history. In percentage terms, this applies to 35% of our participants. Furthermore, the recidivism rate of this group of individuals can be classified into the so-called five-category model of (42), for which Eher et al. (37) specified specific recidivism rates for German-speaking countries. If one classifies the recidivism rate of the subgroup of our sample into the data on pedosexual offenses, one will have to assume level IVa, which semantically corresponds to an above-average risk. In this respect, for this subgroup, at least the critique that it is a group of persons without substantial risk seems untenable. Thus, for this group in particular, society would have to provide similar treatment resources as for persons already convicted.

Finally, it is noteworthy that the sample also includes two individuals who first exhibited problematic sexual behavior during the time-at-risk period: one individual committed CSA for the first time, while one participant viewed CSAM for the first time. In principle, it is conceivable that this could have been an iatrogenic effect, as discussed by Beier et al. (6) for the rate of first offenses in their sample. However, in the CSA case according to the documentation, the therapists had considerable doubts about the credibility of the participant’s statement due to psychotic symptoms. In the CSAM case, the participant reported that he saw posing images on a social media platform, but did not search for CSAM. Based on this information, we therefore do not assume an iatrogenic effect.

## 5 Limitations

Our study has numerous limitations: our results are obtained from individuals who have turned to the network *do not offend*. Individuals with undetected CSA or CSAM, and without contact to the network’s offer, may be a different clientele. In this respect, the rates reported here may not be representative of such a sample.

A major limitation of our study is our sample size. According to a recommendation by Vergouwe et al. (43) and Scurich and Krauss (44), there should be approximately 100 events and 100 non-events for stable estimates. However, our study falls well short of this standard with a total of 9 CSA events and 53 CSAM events. In this respect, there is a risk that our inferences are confounded by this problem.

All results are based on subjects’ self-reports. It can be argued that the official data would have led to different and possibly more reliable results since at least in Hamburg the interrater reliability for CSA could not be tested. However, the frequencies of CSA and CSAM based on self-reports in our study are at least as high as in other meta-analyzes that used a criminal record as an outcome measure. In addition, the interrater reliability of CSA was almost perfect in Bamberg, suggesting that the ratings were sufficiently reliable. Nevertheless, our results are still likely to be an underestimation, as not all clients are likely to have admitted to sexual problematic behavior, e.g., for fear of rejection or disappointment of the therapist.

Most persons in our study had treatment in Hamburg or Bamberg, at least proportionally. It is thus possible that the rates reported here would have been different without the accompanying intervention or that the interventions had different effects in Hamburg and Bamberg. For example, it would be conceivable that risk management interventions during the time at risk lowered the actual rate or that, in the sense of an iatrogenic effect (6), the treatment increased the rates. However, under the assumption that at least some individuals in the data of Helmus et al. (16) received treatment as well, the interpretation is more likely that a small number of individuals did not respond to psychotherapy.

The definition of CSAM used here included only photorealistic media, not drawn or animated depictions of child sexual abuse. It is therefore possible that with a comprehensive definition of CSAM, even higher prevalence rates would result. However, we opted for a stricter definition to compare our findings with official crime statistics. In our experience, drawn or animated depictions are much less likely to result in a conviction.

Participants might also have a different understanding of CSAM: For example, some might already classify nudist images accordingly, while others might understand CSAM exclusively as images of child sexual abuse and exclude so-called posing images. In the present study, this problem was addressed by asking professionals to explore participants for specific examples of images and films used. However, it was not verified that professionals adhered to this instruction and participants did not follow their definition of CSAM.

It can be objected that except for the STATIC-C, the instruments studied here were not applied in the way described in their manuals. For example, we were hardly able to draw on third-party documents when rating the instruments. A criminal record was generally not available. In addition, the instruments are designed for individuals with a conviction with CSA or CSAM, which does not apply to many individuals in our sample.

The validity according to which detected and undetected CSA/CSAM were distinguished from each other can be questioned. In our experience, clients are often not very precise at self-reporting past convictions or charges. Moreover, prior convictions often represent a stigma in the perception of individuals, which is why the concealment of criminal history seems plausible.

The instruments used here contain only static risk factors. However, according to various authors (14, 45–47), static and dynamic risk factors should be used for comprehensive risk assessment. Future studies should investigate the importance of dynamic factors in non-forensic samples. In addition to improved risk assessment, this could shed light on which problems of non-forensic individuals lead from pedo-hebephilic interest to undetected sexual problematic behavior. Based on preliminary evidence (8), one hypothesis might be that stronger pedo-hebephilic interests (see our discussion regarding the CASIC above) with high resources and well-developed detection evasion skills (13) may be responsible for this, but this should be tested in further studies, differentiating between subgroups of individuals with CSA and CSAM in the history. The STABLE 2007 might be a promising instrument for the assessment of dynamic risk factors (48), which, according to recent publications (49, 50), might have the potential to predict recidivism of both CSA and CSAM. However, a completely different approach might also be helpful. Looking at newer models of sexual (re)offense behavior [e.g., (32)] it is clear that reoffending is a dynamic process that depends on many different variables that interact with each other. The NBM-RSR (40) proposes a very complex interaction of dynamic risk factors that can lead up to a self-sustaining network representing the risk of reoffending. It suggests that not all risk factors are present at the same time. Instead, they are activated and deactivated by different interactions, so that at one point other risk factors might suddenly be relevant. Aside from dynamic risk factors, it includes biological, biographical, and sociocultural variables. So it could be that for our non-forensic clients a solely static approach, which is very often connected and based upon information from the judicial system, might just not be good enough. A more complex way, including biographical and sociocultural information, which is assessed in a therapy setting, might be better suited.

Finally, it can be criticized that our time-at-risk period of approximately 2.5 years is significantly shorter than the follow-up periods in the studies by Helmus et al. (16) or Seto et al. (17). Parallelizing the follow-up periods would possibly have resulted in even higher rates of CSA or CSAM.

## 6 Conclusion

In summary, the present sample can be considered clinically relevant: We found significantly higher rates of CSA/CSAM in the history of our participants compared to a German representative study. Only 10% of our subjects reported neither viewing CSAM nor committing CSA in the past. Regarding CSAM, the recidivism rate of 39% of a subgroup of our subjects (88%) was found to be 11 times higher than a meta-analytically determined recidivism rate of 3% for individuals with a CSAM conviction. Regarding CSA, our results show that the recidivism rate of 14% of a subgroup of our participants (35%) was not significantly different from the meta-analytically determined rate of 15% for individuals with a conviction for a contact sex offense.

To adequately allocate treatment resources according to the risk principle (14), institutions working with individuals with pedo-hebephilia in a non-forensic context (meaning people that might or might not have offended but have not yet been detected or have completed all legal matters, e.g., the setting of the network *do not offend*) might use the CPORT with CASIC rating. Given that 80% of our subjects (see Table 1: CSAM only + Mixed) reported watching CSAM in their history, the CPORT could be applied to the main part of individuals in our sample to assess the risk for future CSAM or CSA. In contrast, the STATIC-C, currently used in the network *do not offend* [see (20)], and the STATIC-99 do not seem to have predictive validity. As a consequence, we recommend replacing the STATIC-C with the CPORT, when used in a setting with non-forensic individuals as long as no other instrument has been validated for this specific purpose. However, this conclusion is limited for two reasons: First, the degree of validation of the CPORT in forensic samples was considered insufficient (44). Therefore its application in a non-forensic context is even more critical and needs further validation. Second, our results showed that the predictive validity of the CPROT with CASIC rating is significant, but with poor discrimination. Therefore, future studies should identify incremental variables that could improve risk assessments in a non-forensic context. According to our descriptive results, a promising variable might be the non-detected occurrence of CSA or CSAM in the past of individuals.

## Data availability statement

The datasets presented in this article are not readily available because of the sensitive nature of the research. Requests to access anonymized datasets should be directed to the corresponding author.

## Ethics statement

The studies involving humans were approved by the Ethics Committee of the Chamber of psychotherapists Hamburg in April 2015 (ID: 02/2015-PTKHH) and by the Ethics Committee of the University of Bamberg (initial approvement 29 October 2019, revised and extended version approved 30 July 2022). The studies were conducted in accordance with the local legislation and institutional requirements. Written informed consent for participation in this study was provided by the participants’ legal guardians/next of kin.

## Author contributions

FvF: Writing – original draft, Writing – review & editing. RB-K: Writing – original draft, Writing – review & editing. SS: Writing – original draft, Writing – review & editing. JSP: Writing – original draft, Writing – review & editing. JHP: Writing – review & editing. GH: Writing – review & editing. PB: Writing – review & editing.
