# Corrigendum: Individuals under voluntary treatment with sexual interest in minors: what risk do they pose?

**DOI:** 10.3389/fpsyt.2024.1408862

**Published:** 2024-04-26

**Authors:** Fritjof von Franqué, Ralf Bergner-Koether, Stefanie Schmidt, Jan S. Pellowski, Jan H. Peters, Göran Hajak, Peer Briken

**Affiliations:** ^1^ Institute for Sex Research, Sexual Medicine and Forensic Psychiatry, University Medical Center Hamburg-Eppendorf, Hamburg, Germany; ^2^ Department for Sexual Medicine, Sozialstiftung Bamberg, Bamberg, Germany; ^3^ Department of Clinical Psychology and Psychotherapy, University of Bamberg, Bamberg, Germany; ^4^ Department of Educational Psychology, University of Bamberg, Bamberg, Germany

**Keywords:** child sexual abuse, child pornography, Dunkelfeld, *do not offend*, not become an offender, kein täter werden, CPORT

In the published article, there was an error in **1 Introduction**, Paragraph 3. We cited an author who felt misinterpreted by our presentation. The sentence previously stated:

“In addition, it was argued that a great deal of money was being invested in a group of people who might not pose any substantial risk for reoffending (12).”

The corrected text appears below:

“In addition, König (2015, p.119, translated from the original German work) (9) argued: ‘Instead of expanding primary preventive services, the effectiveness of which has yet to be proven in terms of child and youth protection, it would make sense from a forensic perspective to expand the outpatient psychotherapeutic care situation for men who have been convicted of sexual offenses against minors and have received court orders for therapy, for example. This is a group of offenders who have already proven their dangerousness and, in accordance with the risk-needs-responsivity principle, have a particular need for help.’”

In the published article there was an error in **4 Discussion**, Paragraph 2. We erroneously cited a personal communication and three references. The sentence previously stated:

“However, critics might argue that resources for treatment should be based on the potential for re-offending and not solely on past offending behaviors, implying that many individuals in the prevention project would not offend a second time [H.-L. Kröber, personal communication, January 9th, 2015; (9, 10, 12)].”

The corrected sentence appears below:

“However, critics might argue that resources for treatment should be based on the potential for re-offending and not solely on past offending behaviors, implying that many individuals in the prevention project would not offend a second time.”

In the published article there was a rounding error in a **Table 2**. In the row *Total (*n *= 165)*, 9 out of 165 people are incorrectly reported as 6%. The correct relative frequency is 5%. The corrected **Table 2** appears below:

**Table 2:** Frequencies (percent within group) of child sexual abuse (CSA) during time at risk, differentiated by detection of CSA offending in history.

CSA in history	No offence (n = 107)	1 (1%)
	Undetected CSA (n = 46)	8 (17%)
	Detected CSA (n = 12)	0 (0%)
Total (n=165)		9 (5%)

The authors apologize for these errors and state that this does not change the scientific conclusions of the article in any way. The original article has been updated.

