# Corrigendum: Individuals under voluntary treatment with sexual interest in minors: what risk do they pose?

**DOI:** 10.3389/fpsyt.2025.1608123

**Published:** 2025-06-18

**Authors:** Fritjof von Franqué, Ralf Bergner-Koether, Stefanie Schmidt, Jan S. Pellowski, Jan H. Peters, Göran Hajak, Peer Briken

**Affiliations:** ^1^ Institute for Sex Research, Sexual Medicine and Forensic Psychiatry, University Medical Center Hamburg-Eppendorf, Hamburg, Germany; ^2^ Department for Sexual Medicine, Sozialstiftung Bamberg, Bamberg, Germany; ^3^ Department of Clinical Psychology and Psychotherapy, University of Bamberg, Bamberg, Germany; ^4^ Department of Educational Psychology, University of Bamberg, Bamberg, Germany

**Keywords:** child sexual abuse, child pornography, Dunkelfeld, *do not offend*, not become an offender, kein täter werden, CPORT

In the published article, there were errors.

A correction has been made to **Introduction**, paragraph 3, page 2.

This sentence previously stated:

“In addition, König (12, p.119, translated from the original German work) (9) argued:”

The corrected sentence appears below:

“In addition, König (12, p.119, translated from the original German work) argued:”

A correction has been made to the **Introduction**, paragraph 3, page 2.

This sentence previously stated:

“This is a group of offenders who have already proven their dangerousness and, in accordance with the riskneeds-responsivity principle, have a particular need for help.”

This corrected sentence appears below:

“This is a group of offenders who have already proven their dangerousness and, in accordance with the RNR-principle, have a particular need for help.”

A correction has been made to **Method**, *2.2.1 STATIC-C*, paragraph 1, page 4.

This sentence previously stated: “The items contain the client’s age (1 = older than 25 years), relationship history (1 = never in a relationship of 2 years), non-sexual violence (1 = at least one reported incident of actual, attempted, or threatened harm to another person), reported prior convictions, ICD-10 diagnosis of pedo-hebephilic disorder (1 = pedo-hebephilic disorder, non-exclusive type, 2 = pedo-hebephilic disorder, exclusive type), other paraphilic disorders (1 = paraphilic disorder except sadistic disorder, 2 = sadistic disorder) and personality disorders (1 = any personality disorder) as well as the number (1 = two different persons, 2 = three different persons, 3 = four or more different persons) and sex (1 = male) of individuals harmed plus their relationship (1 = strangers to each other; 1 = unrelated) with the person being assessed.”

The corrected sentence appears below:

“The items contain the client’s age (1 = older than 25 years), relationship history (1 = never in a relationship of 2 years), non-sexual violence (1 = at least one reported incident of actual, attempted, or threatened harm to another person), reported prior convictions, ICD-10 diagnosis of pedo-hebephilic disorder (1 = pedo-hebephilic disorder, non-exclusive type, 2 = pedo-hebephilic disorder, exclusive type), other paraphilic disorders (1 = paraphilic disorder except sadistic disorder, 2 = sadistic disorder) and personality disorders (1 = any personality disorder), prior use of CSAM (1 = prior use of CSAM), as well as the number (1 = two different persons, 2 = three different persons, 3 = four or more different persons) and sex (1 = male) of individuals harmed plus their relationship (1 = strangers to each other; 1 = unrelated) with the person being assessed.”

A correction has been made to **Method**, *2.2.3 CPORT*, paragraph 2, page 4.

This sentence previously stated: “According to a study by Seto and Eke (32), CPORT score was a moderately strong predictor of any sexual recidivism (AUC = 0.74, 95% CI = 0.63, 0.84) and of any child pornography recidivism (AUC = 0.76, 95% CI = 0.65, 0.88).”

This corrected sentence appears below:

“According to a study by Seto and Eke (19), CPORT score was a moderately strong predictor of any sexual recidivism (AUC = 0.74, 95% CI =0.63, 0.84).”

A correction has been made to **Method**, *2.6 Statistics*, paragraph 1, page 6.

This sentence previously stated “For the calculation of the relative risk, we took the data on the total sample and the absolute frequency of CSA/CSAM for the expected frequencies from the respective publications of Dombert et al. (3), Helmus et al. (16) or Seto et al. (17).”

The corrected sentence appears below:

“For the calculation of relative risks, we used the unrounded probabilities from our sample and from the corresponding publications by Dombert et al. (3), Helmus et al. (16) or Seto et al. (17), whereby we also calculated confidence intervals in the case of available absolute values.”

A correction has been made to **Results**, *3.2 CSA and CSAM during time at risk*, paragraph 2, page 6.

This sentence previously stated: “According to the exact binomial test, there was no statistical difference between the observed frequency of CSA of 0.14 and the expected frequency of 0.15, *p* < 0.001 (1-sided). A relative risk of 0.92 (95% CI = 0.483, 1.75) resulted.”

The corrected sentence appears below:

“According to the exact binomial test, there was no statistical difference between the observed frequency of CSA of 0.14 and the expected frequency of 0.15, *p* = 0.49 (1-sided). A relative risk of 0.92 (95% CI not available) resulted.”

A correction has been made to **Results**, *3.2 CSA and CSAM during time at risk*, paragraph 4, page 6.

This sentence previously stated:

“In the exact binomial test, a significant difference between the expected frequency of 0.034 and the observed frequency of 0.39 (*p* < 0.001, 1-sided) with a relative risk of 11.7 (95% CI =8.1; 16.8) resulted.”

The corrected sentence appears below:

“In the exact binomial test, a significant difference between the expected frequency of 0.034 and the observed frequency of 0.39 (*p* < 0.001, 1-sided) with a relative risk of 11.42 (95% CI =7.95, 16.41) resulted.”

A correction has been made to **Results**, *3.3.1 Risk for CSA*, paragraph 1, page 7.

This sentence previously stated:

“Analyzes including all participants revealed no significant association between CSA during time at risk with the total sum scores of the STATIC-99 (r = 0.06, *p* = 0.48), the STATIC-C (r = 0.08, *p* = 0.33), the CPORT with clinical diagnosis (r = 0.09, *p* = 0.27) and the CPORT with CASIC rating (r = 0.08, *p* = 0.32).”

The corrected sentence appears below:

“Analyzes including all participants revealed no significant association between CSA during time at risk with the total sum scores of the STATIC-99 (r = 0.06, *p* = 0.48), the STATIC-C (r = 0.07, *p* = 0.34), the CPORT with clinical diagnosis (r = 0.09, *p* = 0.27) and the CPORT with CASIC rating (r = 0.08, *p* = 0.32).”

A correction has been made to **Results**, *3.3.1 Risk for CSA*, paragraph 2, pages 7-8.

This paragraph previously stated:

“When restricting analyzes to the 58 participants with a history of CSA (see **Table 1**: CSA only + Mixed), no significant association resulted between CSA recidivism with the total sum scores of the STATIC-99 (r = −0.03, *p* = 0.84) or the STATIC-C (r = −0.07, *p* = 0.60). Accordingly, the AUC values from the STATIC-C (AUC = 0.47, 95% CI = 0.25, 0.69, *p* = 0.77) and the STATIC-99 (AUC = 0.49, 95% CI = 0.29, 0.69, *p* = 0.95) were not significantly different from 0.50.”

The corrected paragraph appears below:

“When restricting analyzes to the 58 participants with a history of CSA (see **Table 1**: CSA only + Mixed), no significant association resulted between CSA recidivism with the total sum scores of the STATIC-99 (r = −0.03, *p* = 0.84) or the STATIC-C (r = −0.07, *p* = 0.59). Accordingly, the AUC values from the STATIC-C (AUC = 0.46, 95% CI = 0.25, 0.68, *p* = 0.74) and the STATIC-99 (AUC = 0.49, 95% CI = 0.29, 0.69, *p* = 0.95) were not significantly different from 0.50.”

A correction has been made to **Results**, *3.3.1 Risk for CSA*, paragraph 3, page 8.

These sentences previously stated:

“When restricting analyzes to the 132 participants with a history of CSAM (see **Table 1**: CSAM only + Mixed), no significant correlation resulted between future CSA with the total sum scores of the CPORT with clinical diagnosis (r = 0.10, *p* = 0.26) or the CPORT with CASIC rating (r = 0.10, *p* = 0.28). However in the ROC-Analysis, there was a significant association between future CSA and CPORT with CASIC rating (AUC = 0.69, 95% CI = 0.55, 0.82, *p* = 0.006), but not with the CPORT with clinical diagnosis (AUC = 0.68, 95% CI = 0.50, 0.85, *p* = 0.054).”

The corrected sentence appears below:

“When restricting analyzes to the 132 participants with a history of CSAM (see **Table 1**: CSAM only + Mixed), no significant correlation resulted between future CSA with the total sum scores of the CPORT with clinical diagnosis (r = 0.10, *p* = 0.26) or the CPORT with CASIC rating (r = 0.09, *p* = 0.28). However in the ROC-Analysis, there was a significant association between future CSA and CPORT with CASIC rating (AUC = 0.69, 95% CI = 0.55, 0.82, *p* = 0.006), but not with the CPORT with clinical diagnosis (AUC = 0.68, 95% CI = 0.50, 0.86, *p* = 0.05).”

A correction has been made to **Results**, *3.3.1 Risk for CSA*, paragraph 1, page 8.

These sentences previously stated:

“Analyzes including all participants revealed no significant associations between future CSAM during time at risk and the total sum score of the STATIC-C (r = 0.13, *p* = 0.10) or STATIC-99 (r = 0.06, *p* = 0.48), but significant associations between future CSAM and the total sum score of the CPORT with clinical diagnosis (r = 0.20, *p* = 0.01) and the CPORT with CASIC rating (r = 0.27, *p* < 0.001). According to the ROC-Analysis, the STATIC-C (AUC = 0.60, 95% CI = 0.51, 0.69, *p* = 0.03), the CPORT with clinical diagnosis (AUC = 0.61, 95% CI = 0.51, 0.70, p = 0.03) and the CPORT with CASIC rating (AUC = 0.66, 95% CI = 0.57, 0.75, *p* = 0.001) were able to predict future CSAM, but not the STATIC-99 (AUC = 0.56, 95% CI = 47, 65, *p* = 21).”

The corrected sentences appear below:

“Analyzes including all participants revealed no significant associations between future CSAM during time at risk and the total sum score of the STATIC-C (r = 0.13, *p* = 0.11) or STATIC-99 (r = 0.06, *p* = 0.48), but significant associations between future CSAM and the total sum score of the CPORT with clinical diagnosis (r = 0.20, *p* = 0.01) and the CPORT with CASIC rating (r = 0.27, *p* < 0.001). According to the ROC-Analysis, the STATIC-C (AUC = 0.60, 95% CI = 0.51, 0.69, *p* = 0.04), the CPORT with clinical diagnosis (AUC = 0.61, 95% CI = 0.51, 0.70, *p* = 0.03) and the CPORT with CASIC rating (AUC = 0.66, 95% CI = 0.57, 0.75, *p* = 0.001) were able to predict future CSAM, but not the STATIC-99 (AUC = 0.56, 95% CI = 0.47, 0.65, *p* = 0.21).”

A correction has been made to **Results**, *3.3.2 Risk for CSAM*, paragraph 2, page 8.

These sentences previously stated “When the analyzes were restricted to 132 participants with a history of CSAM (see **Table 1**: CSAM only + Mixed), no significant correlation resulted between future CSAM with the total sum scores of the STATIC-C (r = 0.05, *p* = 0.57) and the CPORT with clinical diagnosis (r = 0.16, *p* = 0.08), but with the CPORT with CASIC rating (r = 0.24, *p* = 0.006). In the ROC-Analysis, there was a significant association between CSAM recidivism and the CPORT with CASIC rating (AUC = 0.63, 95% CI = 0.53, 0.73, *p* = 0.009), but not with the STATIC-C (AUC = 0.55, 95% CI = 0.45, 65, *p* = 0.35) or the CPORT with clinical diagnosis (AUC = 0.57, 95% CI = 0.46, 0.67, *p* = 0.052).”

The corrected sentences appear below:

“When the analyzes were restricted to 132 participants with a history of CSAM (see **Table 1**: CSAM only + Mixed), no significant correlation resulted between future CSAM with the total sum scores of the STATIC-C (r = 0.04, *p* = 0.62) and the CPORT with clinical diagnosis (r = 0.16, *p* = 0.08), but with the CPORT with CASIC rating (r = 0.24, *p* = 0.006). In the ROC-Analysis, there was a significant association between CSAM recidivism and the CPORT with CASIC rating (AUC = 0.63, 95% CI = 0.53, 0.73, *p* = 0.009), but not with the STATIC-C (AUC = 0.55, 95% CI = 0.45, 0.65, *p* = 0.32) or the CPORT with clinical diagnosis (AUC = 0.57, 95% CI = 0.46, 0.67, *p* = 0.22).”

A correction has been made to **Results**, *3.3.2 Risk for CSAM*, paragraph 3, page 8.

These sentences previously stated:

“When restricting the analysis to the 58 participants with a history of CSA (see **Table 1**: CSA only + Mixed), no significant association resulted between future CSAM and the total sum scores of the STATIC-99 (r = −0.02, *p* = 0.91) or the STATIC-C (r = 0.18, *p* = 0.18). According to the AUC-Values, there was no significant association between future CSAM and the STATIC-C (AUC = 0.60, 95% CI = 0.45, 0.75, *p* = 0.18) or the STATIC-99 (AUC = 0.52, 95% CI = 0.37, 0.67, *p* = 0.79).”

The corrected sentences appear below:

“When restricting the analysis to the 58 participants with a history of CSA (see **Table 1**: CSA only + Mixed), no significant association resulted between future CSAM and the total sum scores of the STATIC-99 (r = −0.02, *p* = 0.91) or the STATIC-C (r = 0.17, *p* = 0.20). According to the AUC-Values, there was no significant association between future CSAM and the STATIC-C (AUC = 0.60, 95% CI = 0.45, 0.75, *p* = 0.20) or the STATIC-99 (AUC = 0.52, 95% CI = 0.37, 0.67, *p* = 0.79).”

A correction has been made to **Discussion**, paragraph 2, page 9.

The sentence previously stated:

“There is currently a lack of empirical criteria to distinguish individuals with an increasing profile from those with a consistently low risk, who, according to the RNR-model, require further treatment or either do not need treatment at all, or should receive little treatment (41)”

The corrected sentence appears below:

“There is currently a lack of empirical criteria to distinguish individuals with an increasing profile from those with a consistently low risk, who, according to the RNR-model, either do not need treatment at all, or should receive little treatment (41).”

A correction has been made to **Discussion**, paragraph 2, page 9.

The sentence previously stated:

“However, from a risk perspective and regarding the moderate predictive validity of the CPORT with CASIC rating, one would additionally wish for a measure with CSAM stronger associations with the CSAM recidivism rate.”

The corrected sentence appears below:

“However, from a risk perspective and regarding the moderate predictive validity of the CPORT with CASIC rating, one would additionally wish for a measure with stronger associations with the CSAM recidivism rate.”

There was an error in **Figure 1** “Inclusion process of the study”, page 3.

Instead of “HH = 238” excluded persons due to diagnostic or insufficient data, it should be “HH = 240”.

The corrected **Figure 1** appears below.

**Figure 1:**
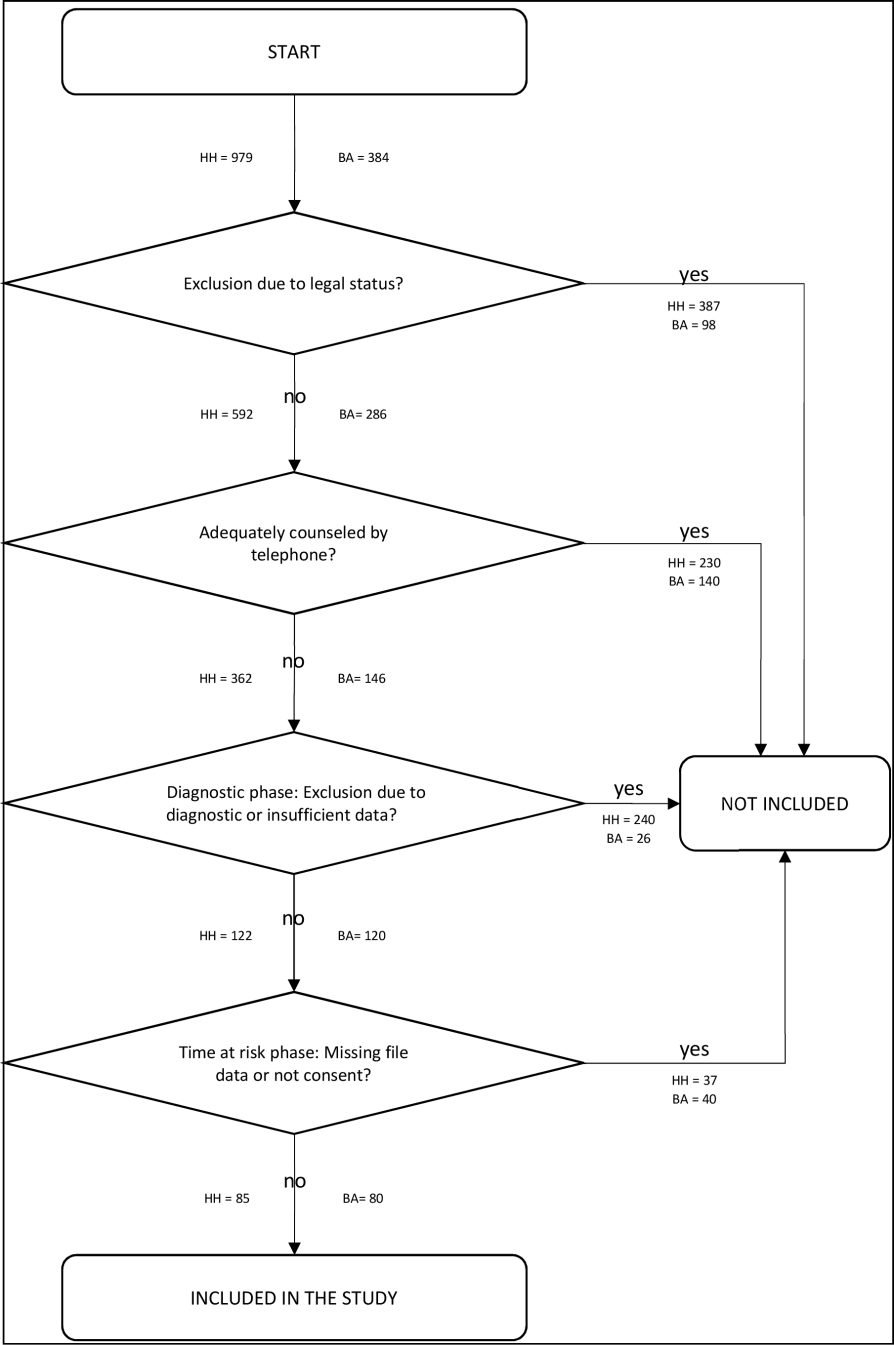
Inclusion process of the study. HH, HAMBURG/Germany; BA, BAMBERG/Germany.

There were errors in **Table 1** “Descriptive data from 165 non-forensic clients with sexual interest in children, distinguished by sexual problem behaviors in their history”, page 7.

The corrected **Table 1** appears below.

**Table 1:** Descriptive data from 165 non-forensic clients with sexual interest in children, distinguished by sexual problem behaviors in their history.

Mean (SD)/Number(Percent)/Median (Range)	GROUP				
	No offence (n=17)	CSAM^a^ only (n=90)	CSA^b^ only (n=16)	Mixed^c^ (n=42)	Total (n=165)
Sociodemographic data					
age in years (SD)	31.06 (7.90)	34.21 (11.56)	44.44 (16.05)	39.21 (10.76)	36.15 (12.04)
male subjects	15 (88%)	89 (99%)	16 (100%)	42 (100%)	162 (98%)
more than 10 years in school	10 (59%)	67 (74%)	8 (50%)	26 (62%)	111 (67%)
with a job	11 (65%)	65 (72%)	11 (69%)	31 (74%)	118 (72%)
with an intimate relationship	4 (24%)	39 (43%)	9 (56%)	26 (62%)	78 (47%)
with children	2 (12%)	19 (21%)	8 (50%)	20 (48%)	49 (30%)
living alone	7 (41%)	44 (49%)	8 (50%)	14 (33%)	73 (44%)
Diagnostic data					
with ICD-10 pedophilic disorder	10 (59%)	84 (93%)	9 (56%)	42 (100%)	145 (88%)
with CASIC-Score >2	2 (12%)	48 (53%)	3 (19%)	21 (50%)	74 (45%)
with ICD-10 pedophilic disorder and CASIC- Score >2	2 (12%)	45 (50%)	2 (13%)	21 (50%)	70 (42%)
with any other ICD-10 paraphilia	4 (24%)	16 (18%)	5 (31%)	11 (26%)	36 (22%)
with hypersexual disorder	1 (6%)	10 (11%)	0 (0%)	2 (5%)	13 (8%)
with any personality disorder	4 (24%)	12 (13%)	3 (19%)	9 (21%)	28 (17%)
with any affective disorder	2 (12%)	30 (33%)	1 (6%)	10 (24%)	43 (26%)
Forensic data					
Previous conviction for CSAM	0 (0%)	14 (16%)	0 (0%)	7 (17%)	21 (13%)
Previous conviction for CSA	0 (0%)	0 (0%)	4 (25%)	8 (19%)	12 (7%)
Risk-Assessment data					
months at risk (SD)	22.70 (23.79)	28.47 (24.30)	23.90 (20.21)	28.32 (22.21)	27.40 (23.25)
STATIC-99 score (SD)	1.18 (.64)	1.21 (1.11)	1.69 (1.96)	2.10 (1.43)	1.48 (1.31)
STATIC-99 MEDIAN (RANGE)	1.00 (2.00)	1.00 (4.00)	1.00 (5.00)	2.00 (7.00)	1.00 (7.00)
STATIC-C score (SD)	2.65 (1.84)	3.48 (1.66)	3.94 (2.86)	6.02 (2.07)	4.08 (2.24)
STATIC-C MEDIAN (RANGE)	3.00 (8.00)	3.00 (10.00)	3.00 (9.00)	6.00 (9.00)	4.00 (11.00)
CPORT score (SD)	1.65 (1.22)	2.19 (1.04)	1.81 (1.38)	2.45 (1.15)	2.16 (1.14)
CPORT MEDIAN (RANGE)	1.00 (4.00)	2.00 (5.00)	1.50 (5.00)	2.00 (4.00)	2.00 (5.00)
Sexual problematic behaviors during time at risk					
child sexual abuse material	0 (0%)	34 (38%)	1 (6%)	18 (43%)	53 (32%)
child sexual abuse	1 (6%)	0 (0%)	3 (19%)	5 (12%)	9 (5%)

^a^Participants with Child Sexual Abuse Material in their past, ^b^Participants with Child Sexual Abuse in their past, ^c^Participants with Child Sexual Abuse and Child Sexual abuse Material in their past

In the line “With a job”, an incorrect frequency of “11 (63%)” was reported for the no offense group. Instead, it should be “11 (65%)”.

In the line “with intimate relationship”, an incorrect frequency of “19 (56%)” was reported. Instead, it should be “9 (56%)”.

In the line “months at risk”, incorrect numbers were reported for the no offense group (“22.69 (23.79)”) and the total sample (“22.69 (23.79)”). Instead, it should be “22.70 (23.79)” for the no offense group and “27.40 (23.25)” for the total sample.

In the line “STATIC-99 score (SD)”, an incorrect standard deviation for the total sample (“1.48 (1.30)”) was reported. Instead, it should be “1.48 (1.31)”.

In the line “STATIC-99 median (range)”, an incorrect range was reported for the no offense group (“1.00 (1.00)”) and the mixed group (“2.00 (4.00)”). Instead, it should be “1.00 (2.00)” for the no offense group and “2.00 (7.00)” for the mixed group.

In the line “STATIC-C score (SD)”, incorrect values were reported for the CSAM only group (“3.47 (1.66)”), the mixed group (“6.00 (2.06)”) and the total sample (“4.07 (2.24)”). Instead, it should be “3.48 (1.66)” for the CSAM only group, “6.02 (2.07)” for the mixed group and “4.08 (2.24)” for the total sample.

The authors apologize for these errors and state that this does not change the scientific conclusions of the article in any way. The original article has been updated.

